# A case of early diagnosis of pulmonary capillary hemangiomatosis in a worker with exposure to silica

**DOI:** 10.1186/s12890-019-0896-5

**Published:** 2019-07-23

**Authors:** Chang Dong Yeo, Deokjae Han, Jongmin Lee, Woo-Baek Chung, Jung Im Jung, Kyo-Young Lee, Tae-Jung Kim, Woori Jang, Myungshin Kim, Ji Young Kang

**Affiliations:** 1 Division of Allergy and Pulmonology, Department of Internal Medicine, College of Medicine, Seoul St. Mary’s Hospital, The Catholic University of Korea, 222 Banpo-daero, Seocho-gu, Seoul, 06591 Republic of Korea; 2 Department of Radiology, College of Medicine, The Catholic University of Korea, Seoul, Republic of Korea; 3 Department of Clinical Pathology, College of Medicine, The Catholic University of Korea, Seoul, Republic of Korea; 4 Department of Laboratory Medicine, College of Medicine, The Catholic University of Korea, Seoul, Republic of Korea; 5 Catholic Genetic Laboratory Center, College of Medicine, The Catholic University of Korea, Seoul, Republic of Korea

**Keywords:** Pulmonary capillary hemangiomatosis, Pulmonary hypertension, Silica or organic solvent

## Abstract

**Background:**

Pulmonary capillary hemangiomatosis (PCH) is a progressive and refractory vascular disease in the lung. Pulmonary hypertension is frequently combined with PCH when capillary proliferation invades to nearby pulmonary vascular systems. It is difficult to differentiate PCH from other diseases such as pulmonary venoocclusive disease and pulmonary arterial hypertension that cause pulmonary hypertension as they frequently overlap.

**Case presentation:**

A 29-year-old female who had worked at a bathtub factory presented with progressive exertional dyspnea for the past 2 years. Computed tomography revealed centrilobular, diffusely spreading ground-glass opacities sparing subpleural parenchyma with some cystic lesions and air-trapping in both lungs, suggesting a peculiar pattern of interstitial lung disease with airway involvement. There was not any evidence of right heart failure or pulmonary hypertension on echocardiogram, as well as radiography. Microscopic examination of the lung by thoracoscopic resection showed atypical proliferation of capillary channels within alveolar walls and interlobar septa, without invasion of large vessels.

**Conclusion:**

We experienced a pathologically diagnosed PCH in a young female complaining progressive dyspnea with prior exposure to occupational silica or organic solvent without elevated right ventricular systolic pressure (RVSP) who showed atypical pattern of radiologic findings.

## Background

Pulmonary capillary hemangiomatosis (PCH) is a rare and refractory capillary proliferation within the alveolar septae. This disease is characterized by a progressive clinical course with signs and symptoms of hypoxemia, alveolar hemorrhage, right heart failure, and pulmonary hypertension [1, 2]. PCH has been described in patients ages 2 to 71 years [3]. Pivotal diagnostic histologic feature is the proliferation of capillaries in the pulmonary interstitium that invade into nearby structures such as pulmonary vein or arteries, alveolar wall, interlobular septa, and bronchi. Affected areas tend to have a patch distribution within the lung. Reticulonodular infiltrates on radiography or diffuse centrilobular ground-glass opacities on computed tomography (CT) scan are typical findings of PCH [2, 4]. Definite treatment is bilateral lung transplantation. PCH has poor prognosis with a median survival of 3 years after initial clinical symptom without transplantation [3].

Most cases of PCH have been accompanied with pulmonary hypertension except a few reports [5]. Furthermore, clinical manifestations and radiographic features of PCH are similar to those of other causes of pulmonary hypertension such as pulmonary veno-occlusive disease (PVOD) or idiopathic pulmonary artery hypertension [6]. In particular, PCH and PVOD frequently share common findings including septal lines, lymph node enlargements, pulmonary artery dilatation, pericardial effusion, and pleural effusion in high resolution CT (HRCT) due to hemodynamic derangements and underlying capillary or postcapillary disorder [7]. Clinical differential diagnosis of above diseases has not been established. Prostacyclin therapy for pulmonary hypertension could aggravate pulmonary edema in patient with PCH [4]. Therefore, early recognition for differential diagnosis is important and histological diagnosis should be considered if clinically feasible.

Causes or risk factors of PCH are not clearly identified yet, although some cases have reported its association with connective tissue diseases or as a familial disease [8, 9]. As for PVOD, a few cases have reported the development of the disease following various occupational exposures to organic solvents [10]. However, there have been few reports of PCH associated with exposure to occupational substances. We experienced an interesting case of a young female with progressive breathlessness who was exposed to occupational silica or organic solvent. She showed tiny nodules of ground-glass opacity combined multiple cysts and air-tapping on radiology without elevated right ventricular systolic pressure (RVSP). Therefore, occupational lung disease or interstitial lung disease was clinically suspected. However, it was finally diagnosed as PCH by video-associated thoracoscopic lung biopsy.

### Case presentation

**Fig. 1:**
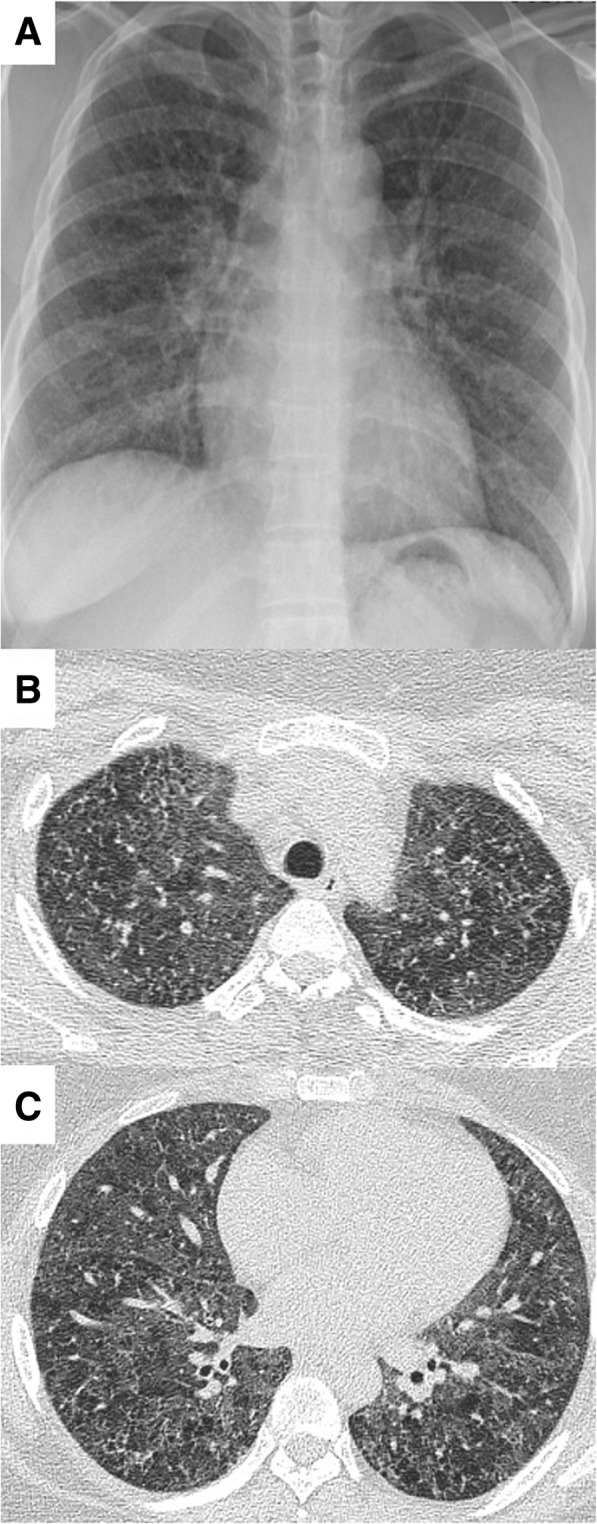
Imaging findings of the chest in the patient at hospital admission. **a** Chest X-ray showing diffusely reticulo-nodular densities in the whole lung field. **b** HRCT of the chest revealing central oriented ground glass opacities with some cystic lesions and airway trapping. **c** They had no lobar predominance with sparing subpleural area

**Fig. 2:**
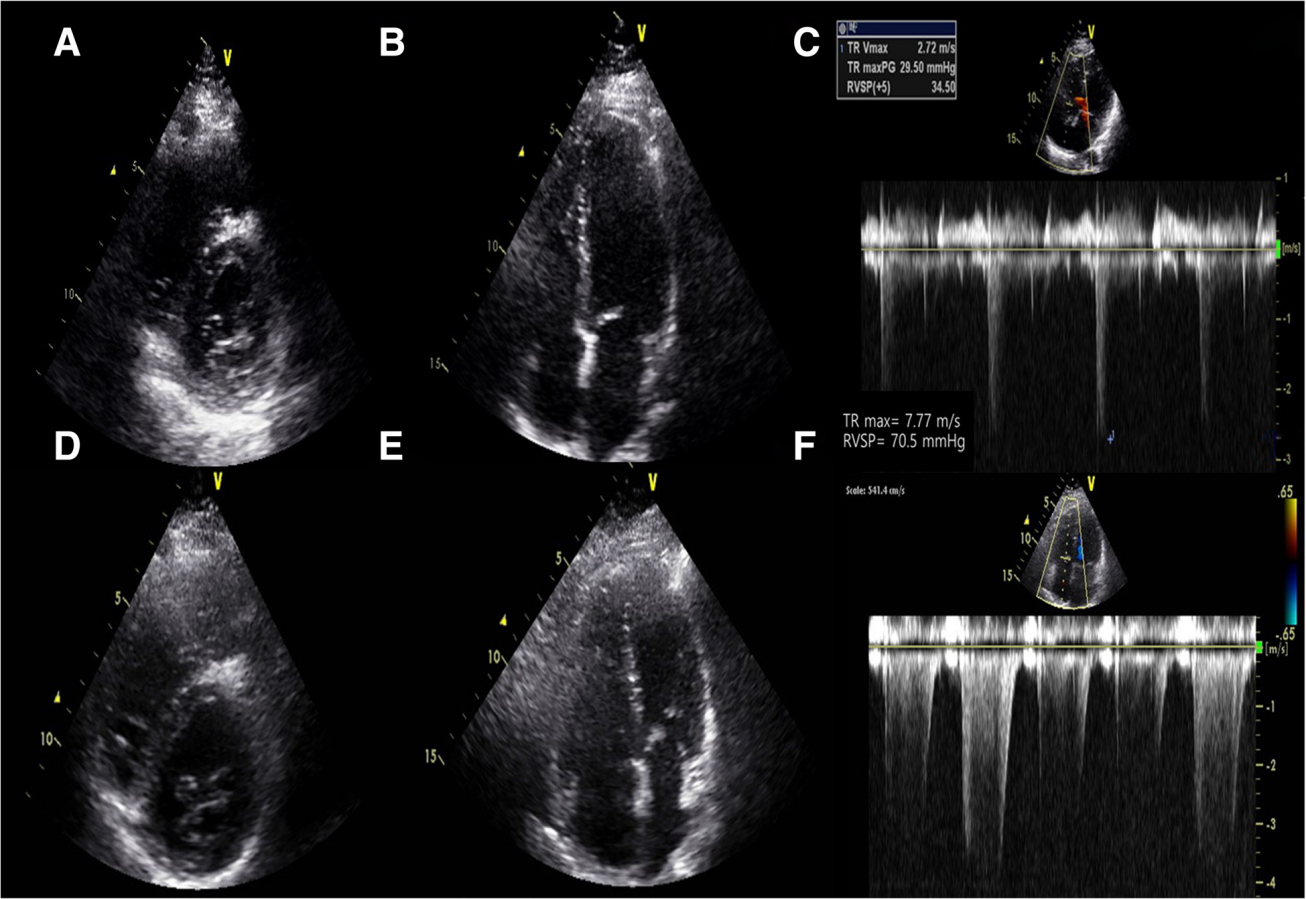
Changes of echocardiogrphic findings with 2.5 years of interval. Parasternal short axis (**a**) and apical 4-chamber (**b**) view at the initial visit demonstrated normal chamber size. Estimated RVSP was 34.5 mmHg (**c**). At the follow-up of 20 months, D-shaped LV (**d**) and RV enlargement (**e**) demonstrated estimated RVSP was 70.5 mmHg (**f**). LV; left ventricle: RV; right ventricle

**Fig. 3:**
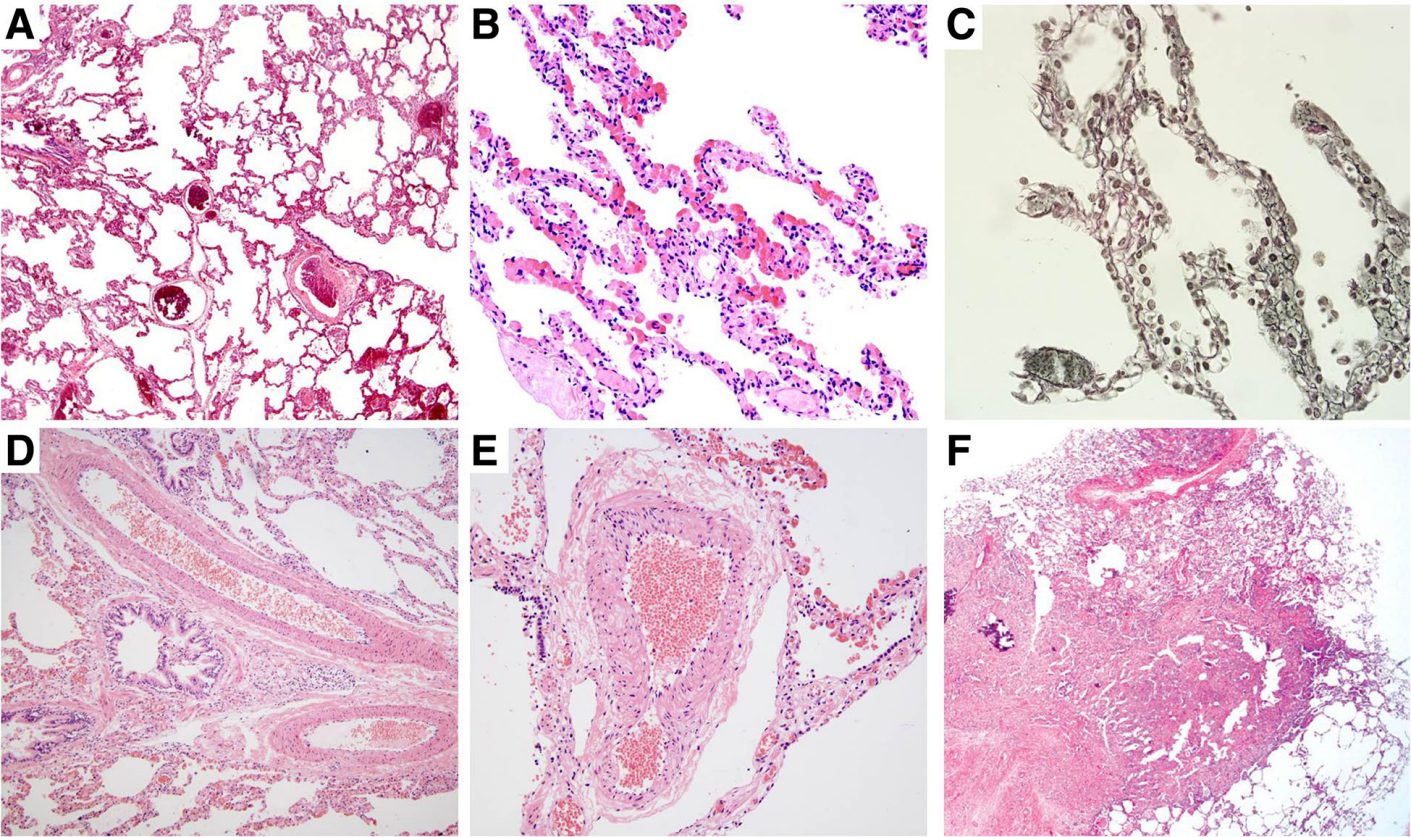
Histologic findings of pulmonary capillary hemangiomatosis. **a** Low-power view showing marked congestion. **b** Higher-power image demonstrating thickened alveolar walls caused by proliferating capillaries (Hematoxilin and eosin staining, X400). **c** Proliferating capillary channels are well demonstrated by reticulin staining (Reticulin, X400). **d** There was no capillary proliferation within walls of the larger vessel or luminal narrowing or obliteration (Hematoxilin and eosin staining, X100). **e** Small arteries showing muscular hypertrophy (Hematoxilin and eosin staining, X200). **f** There are focal fibroses and calcifications (Hematoxilin and eosin staining, X2)

**Fig. 4:**
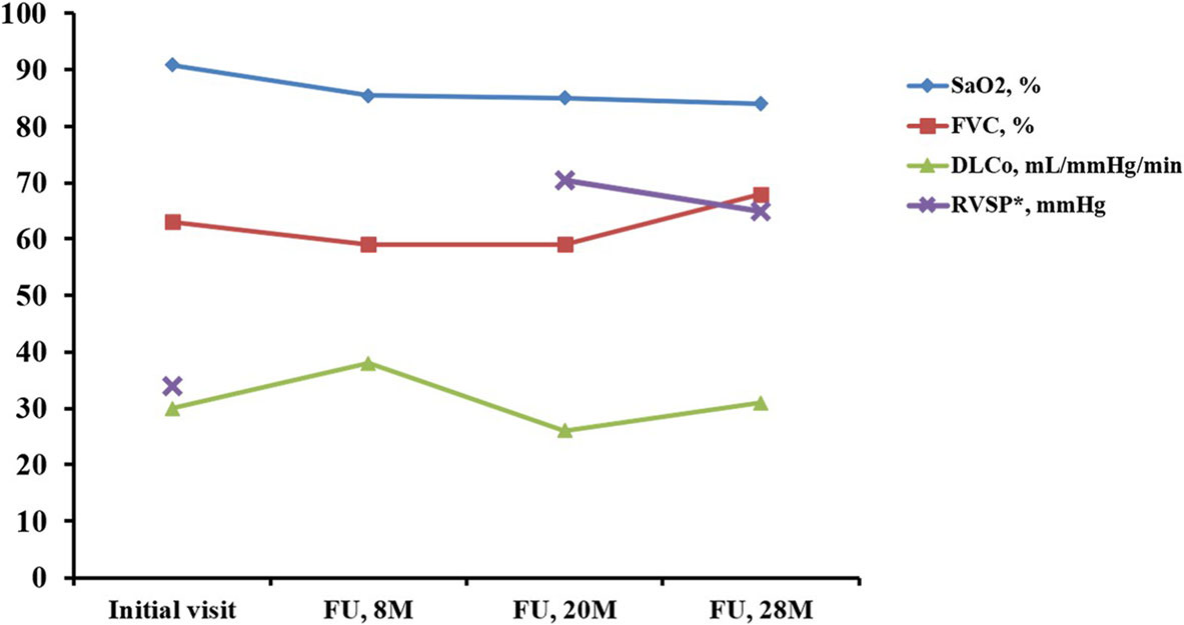
Serial changes of cardiopulmonary parameters in the patient. Over 2.5 years of follow-up, arterial oxygen saturation measured at room air gradually declined. Whereas the level of RVSP at the time of diagnosis with pulmonary capillary hemangiomatosis, was normal limit of 34 mmHg, it was elevated remarkably to 70 mmHg, at the follow-up of 20 months. *RVSP was not measured at the follow- up of 7 months. FU; follow-up, M; month

A 29-year-old female presented with progressive exertional dyspnea for the past 2 years. She had worked at a bathtub factory with constant exposure to silica or various organic solvent (mainly resin detergent and acetone solvent) within the workplace for 2 years until she visited our hospital. She used a simple mask, not a respirator, to protect dust during work. She had no previous history of disease or medication. She had no specific familial disease either. She was an ex-smoker with 10 pack-year with quitting 1 month ago. Her dyspnea was New York Heart Association class III. It was associated with cough. Her blood pressure, heart rate, arterial oxygen tension (PaO_2_), and oxygen saturation on room air were 110/60 mmHg, 90 beats/min, 60.1 mmHg, and 90.8%, respectively. Coarse breathing sounds with rales were heard at the whole lung fields. Chest X-ray showed bilateral reticular nodular shadows (Fig. 1a). HRCT revealed central-dominant, diffusely spreading ground-glass opacities sparing subpleural parenchyma with some cystic lesions and air-trapping in both lungs (Fig. 1b and c). On pulmonary function test, diffusion capacity for carbon monoxide was severely impaired at 30% of the predicted. Her hemoglobin and hematocrit levels were 18.2 g/dL and 52.0%, respectively. Other complete blood counts were normal. Liver and renal function tests were also normal. Serological tests for human immunodeficiency virus, hepatitis B virus, and hepatitis C virus were negative. Autoimmune studies such as rheumatoid factor, anti-nuclear antibody, anti-neutrophil cytoplasmic antibody, and anti-cardiolipin antibody were all nonspecific. Thyroid profile and troponin I were negative. Echocardiography revealed normal size with wall thickness in four chambers, nonspecific appearance on inferior vena cava, and 34 mmHg on RVSP, suggesting no definite evidence of right heart failure (Fig. 2a-c). Thoracoscopic wedge resection at the right middle lobe was performed for histopathologic diagnosis. Grossly, lungs presented multiple, red-brown, ill-defined nodular lesion throughout the whole parenchyma. Microscopic examination of the lung wedge resection showed marked congestion and striking capillary proliferation in alveolar walls well demonstrated by reticulin staining. However, there was no capillary proliferation within walls of the larger vessel. There was no luminal narrowing or obliteration except small arteries showing muscular hypertrophy. There was focal fibrosis accompanied by calcifications (Fig. 3). Mutation test for eukaryotic translation initiation factor 2α kinase 4 (*EIF2AK4*) was negative (Table 1). Taken clinical features and laboratory findings together, PCH was finally diagnosed and she was discharged with home oxygen therapy. During follow up for 2.5 years, her dyspnea slowly aggravated and she developed elevated RVSP 70 mmHg on echocardiography (Fig. 2d-f). Serial changes of cardiopulmonary parameters in the patient were in the Fig. 4. Therefore, she was planned to undergo lung transplantation.

**Table. 1:** Results of *EIF2AK4* gene mutation test

Location	Nucleotide change	Amino acid change	Allelic status	DbSNP ID
Exon1	c.99 T > C	p.=	Homozygous	rs566792
Intron7	c.859 + 55A > G		Heterozygous	rs510949
Exon9	c.1321A > C	p.Ile441Leu	Heterozygous	rs2291627
Exon11	c.1667A > G	p.Glu556Gly	Heterozygous	rs2307105
Intron16	c.2631 + 47 T > C		Homozygous	rs4144472
Intron18	c.2766 + 71A > G		Heterozygous	rs11633992
Intron24	c.3408-14G > C		Heterozygous	rs2279580
Intron30	c.4174-17G > T		Homozygous	rs12916520
Intron34	c.4562-8G > T		Homozygous	rs2250402
Intron36	c.4728 + 43 T > C		Homozygous	rs4432245

All sequence variants are described in reference to RefSeq transcript NM_001013703.3

## Discussion and conclusions

PCH/PVOD is rare. It is the most difficult disease entity to diagnose and treat, especially PCH which is less frequent than PVOD. There have been only fewer than 100 cases of PCH reported to date [4]. It has been suggested that prevalence of PVOD is between 0.3 and 1.4 cases per million persons [11]. Distinguishing PCH or PVOD from other causes of pulmonary hypertension can be challenging for clinicians, radiologists, and pathologists [6]. In addition, since both PCH and PVOD have similar clinical, imaging, and hemodynamic presentations with poor prognosis, differential clinical and pathological diagnosis between the two needs to be guided by physicians with expertise.

Although similar radiologic features are common for both PCH and PVOD, basilar reticulonodular opacities and large sized of ground-grass opacities are seen in the former, but uncommon in the latter [6, 12]. The current case showed diffusely ground glass opacities at both lungs with tiny nodules on chest HRCT. Intriguingly, some peculiar features such as air-trapping, cystic lesions, and focal bronchiolectasis were also observed in the whole lung fields, implying airway-disease pattern, which are atypical image findings in PCH. Clinical characteristics such as young female and occupational history made our clinicians to first consider other diseases including occupational lung disease and interstitial lung diseases. However, she had no symptoms or laboratory findings associated with connective tissue disorder. She did not show elevated RVSP or radiographic evidence of pulmonary hypertension or vascular involvement of PCH. Furthermore, pathologic examination of the lung tissue revealed diffusely thickened alveolar walls resulting from remarkable capillary proliferations and infiltration to the nearby bronchi, consistent with PCH. Histopathological hallmark of PCH is the atypical proliferation of capillary channels within alveolar walls and interlobar septa, which is compatible with our findings [3]. In some previous reports, PCH is accompanied by pulmonary fibrosis or emphysema which induces atypical radiologic findings, like our case [13, 14]. They explained that repeated alveolar hemorrhage or inflammation in PCH might induce secondary pulmonary fibrosis. The present case also showed focal parenchymal fibrosis with calcification in histology. However, there was no massive parenchymal or pleural fibrosis, with which we can rule out the possibilities of silicosis or asbestosis. This could make CT imaging complicated and uncommon, combined with an absence of pulmonary hypertension. She had no clinical symptoms suggesting asthma and allergic diseases. In addition, based on laboratory findings such as eosinophil counts and allergen specific IgE test, and pulmonary function test, there was no evidence of allergic lung disease.

PCH is generally accompanied by pulmonary hypertension, although their relationship has not been elucidated yet. In a report by Xie et al. [15], 78% of 64 PCH cases had pulmonary hypertension. An uncontrolled proliferation of pulmonary capillaries infiltrating vascular, bronchial, and interstitial pulmonary structure could be a plausible explanation. In addition, these proliferating capillaries can surround and compress walls of pulmonary venules and veins, causing intimal fibrosis and secondary veno-occlusion, thus increasing pulmonary vascular resistance in PCH [16]. In the present case, the histology showed capillaries infiltrated the alveolar and bronchial walls, but not the vascular wall. This might have contributed to the maintenance of pulmonary blood flow in the early stage of natural disease course. There are a few PCH cases without pulmonary hypertension characterized by no definite evidence of perivascular or intravascular proliferation of capillaries [5].

As for an occupational history, the patient worked at a bathtub manufacturing factory without proper respiratory protection. She could have been exposed to silica or organic solvent. Considering these special situation and an onset time of dyspnea reminded us of acute silicoproteinosis, one of silica-related respiratory diseases [17]. However, she did not show any typical findings of silicoproteinosis such as bilateral airspace disease with consolidation typically involving posterior portions of lungs on chest CT and Periodic acid-Schiff-positive lipoproteinaceous material filling the air space [18].

Montani et al. [10] have reported that occupational exposure to organic solvents might present a risk factor for PVOD. Although crucial causes of risk factors of PCH have not been identified yet, PCH can be developed secondarily in lungs with PVOD, connective tissue diseases, and chronic passive congestion based on a few case reports [19]. Considering the above relation between PCH and PVOD, occupational exposure to silica or organic solvent in the current patient might have influenced the clinical course of PCH, although whether and to what extent of the exposure might be related to the development of PCH are unclear.

Recently, mutation in *EIF2AK4* has been reported to be one of the causes for autosomal recessive PCH with both familial and sporadic origin [9]. EIF2AK4 expression, or lack thereof, contributes to the regulation of angiogenesis, affecting endothelial proliferation and apoptosis resistance. In the report, *Best* et al. have identified various mutations, including c.3766C > T and c.1153dupG from two patients with familial PCH and c.1392delT, c.860-1G > A, and c.3438C > T in two patients among 10 sporadic PCH patients [9]. Additionally, *EIF2AK4* mutations were detected in cases of familial and sporadic PVOD, presenting a clear link between PCH and PVOD [20]. However, *EIF2AK4* mutation should not be expected to account for all cases of PCH/PVOD. According to the report of Montani et al. [10], 20% of PVOD cases had *EIF2AK4* mutations. It seems that the presence of additional factors such as environmental factors and additional genetic loci can modify the effect of loss-of-function *EIF2AK4* mutation, yielding a phenotype that can range from one predominantly showing features of PVOD to one that predominantly shows features of PCH [6].

We experienced the pathologically diagnosed as PCH in a young female complaining progressive dyspnea who was exposed to occupational silica or organic solvent without elevated RVSP but with unique radiologic findings. Since surgical biopsy frequently is not feasible due to patient’s condition, early recognition of PCH should be based on clinical and radiologic characteristics. Histological proof is required for patients suspected with occupational lung disease if clinical and imaging patterns are atypical.

## Data Availability

All data are contained within the manuscript.

## Abbreviations

CT: Computed tomography
HRCT: High resolution computed tomography
PCH: Pulmonary capillary hemangiomatosis
PVOD: Pulmonary veno-occlusive disease
